# *Helicobacter* *pylori* Eradication Therapies in the Era of Increasing Antibiotic Resistance: A Paradigm Shift to Improved Efficacy

**DOI:** 10.1155/2012/757926

**Published:** 2012-06-19

**Authors:** Sotirios D. Georgopoulos, Vasilios Papastergiou, Stylianos Karatapanis

**Affiliations:** ^1^Department of Gastroenterology, Athens Medical Center of P. Phaliron, P. Phaliron, 17562 Athens, Greece; ^2^Department of Internal Medicine, General Hospital of Rhodes, 85100 Rhodes, Greece

## Abstract

With the rising prevalence of antimicrobial resistance, the eradication rates of *Helicobacter pylori (H. pylori)* with standard treatments are decreasing to unacceptable levels (i.e., ≤80%) in most countries. After these disappointing results, several authorities have proposed that infection with *H. pylori* should be approached and treated as any other bacterial infectious disease. This implicates that clinicians should prescribe empirical treatments yielding a per protocol eradication of at least 90%. In recent years several treatments producing ≥90% cure rates have been proposed including sequential therapy, concomitant quadruple therapy, hybrid (dual-concomitant) therapy, and bismuth-containing quadruple therapy. These treatments are likely to represent the recommended first-line treatments in the near future. In the present paper, we are considering a series of critical issues regarding currently available means and approaches for the management of *H. pylori* infection. Clinical needs and realistic endpoints are taken into account. Furthermore, emerging strategies for the eradication of *H. pylori* and the existing evidence of their clinical validation and widespread applicability are discussed.

## 1. Introduction

Infection with *Helicobacter pylori (H. pylori)* is a global health problem affecting 20–50% of the western world's population and up to 80% of the population in developing countries [1, 2]. Presence of *H. pylori *is known to be associated with a wide range of gastrointestinal disorders including peptic ulcer, gastric carcinoma, and mucosa-associated tissue lymphoma, and, thus, ability to reliably eradicate the pathogen is important for managing these diseases [3–6]. Several factors are making infection with* H. pylori *so challenging to treat. These factors include (a) the development of *H. pylori* resistance to antibiotics, (b) the large number of bacteria in the stomach, producing an “inoculum” effect, (c) the protection of the thick gastric mucus gel layer, and (d) the intracellular (and thus inaccessible to antibiotics) location of many bacteria [7–9]. Other factors including presence of multiple strain infection and individual factors such as patient's compliance to treatment, age less than 60, the type of gastritis, and presence of nonulcer dyspepsia, where the eradication rates are lower in comparison with peptic ulcer disease, have been also linked to therapy efficacy [10, 11]. Educating the patient on the importance to take the medication as prescribed, warning in advance on the possibility of adverse events, and therefore obtain the maximum in terms of compliance to treatment poses a major clinical challenge to practicing physicians. 

Historically, a wide spectrum of antimicrobial agents have been shown to be effective against *H. pylori* and successfully used in clinical practice. Most commonly are clarithromycin, amoxicillin, metronidazole, tinidazole, tetracycline, and the fluoroquinolones. As experience in treating the infection was gained, these drugs (and with the addition of an antisecretory agent or bismuth) have been used in different combinations, and developed regimens have been tailored in various parameters (dosage, dosing intervals, duration of treatment) in order to provide the best outcome in terms of efficacy and tolerability. However, despite the continuous efforts made by the digestive disease community (and not by experts in infectious diseases), the optimal empirical treatment remains to be discovered. 

In the present paper we are considering a series of critical issues regarding currently available means and approaches for the management of *H. pylori* infection. Realistic needs are taken into account with particular attention to crucial aspects for clinical practice and the importance for posttreatment testing for cure. Furthermore, emerging strategies for the eradication of *H. pylori* and the existing evidence of their clinical validation and applicability are discussed.

## 2. Empirical Triple Therapies: A Declining Clinical Standard

More than a decade ago, recommended therapies comprising of a proton pump inhibitor (PPI), amoxicillin, and clarithromycin (standard triple therapies) yielded high efficacy, providing eradication rates comparable to those expected for other prevalent bacterial infections such as respiratory and urinary tract infections, gonorrhea, and tuberculosis [12, 13]. Unfortunately, in successive years the eradication rates have fallen considerably with these regimens, in some countries to unacceptably low levels (<80% or even <70%), mainly because of the increasing prevalence of resistance to clarithromycin [8, 14, 15]. The widespread use of clarithromycin for infectious diseases other than *H. pylori *infection represents the main reason for the increasing development of resistance to this antibiotic; this explains the lower prevalence of clarithromycin resistance in Northern (versus Southern) European countries where policy for antibiotic use is more stringent [16]. The progressive decline in the efficacy of first-line treatments was already evident in the first meta-analyses published by the early 2000s and indirectly outlined by the European consensus recommendations (Maastricht 2000 and 2005), initially with the adoption of a “cumulative” approach to treat *H. pylori*, which introduced first- and second-line therapies, and later by the definition of a local/regional threshold of resistance to clarithromycin (15–20%), at which the antibiotic should not be used if culture was not previously performed to assess susceptibility [17–20]. In such cases, a bismuth-containing quadruple therapy (comprising of PPI, bismuth, metronidazole, and tetracycline) is recommended as an alternative empirical treatment, although its efficacy does not seem to exceed that of standard regimen according to some studies and a recent meta-analysis [21–23]. Furthermore, the threshold of resistance to clarithromycin at which triple regimens lose their efficacy seems to be substantially lower than 15–20% and may be 10% or even less [24]. 

Currently, standard triple therapy still remains the most widely recommended first-line treatment option worldwide and even in countries where improved alternative therapeutic options have been developed and sufficiently validated in a clinical setting [25–27]. The situation is similar in Greece: triple therapies represent the backbone of routine clinical practice but their performance is steadily declining during the past 10 years [28–31], in parallel with an increase in the incidence of clarithromycin resistance, reportedly from 6% to 26% [32–34]. 

To be fair, the cumulative efficacy of first and second-line treatments proposed by Maastricht 3, together with sensitivity-directed (re)treatment or administration of 3rd and 4th line rescue therapies (based on levofloxacin and rifampicin, resp.), is nearly approaching 100% [31, 35, 36]. However, for this goal to be achievable, patients must be highly compliant with repeated treatment courses. Necessity to use second-line therapies accounts for 20–30% of patients infected with *H. pylori* (intention to treat (ITT) analysis); even second-line therapy is not enough to eradicate the pathogen in 5–10% of cases [31, 37]. These rates are likely to increase further, as antimicrobial resistance becomes more prevalent worldwide. In that setting, patients may be required to complete more than one (and sometimes 3 or 4) complex treatment courses and therefore be exposed to a significant range of potential side effects which can virtually affect adherence and compromise their quality of life. 

Use of an effective first-line treatment is known to provide a key advantage in the eradication of *H. pylori*, namely, prevention of secondary antibiotic resistance [38]. Much effort has been spent on improving currently recommended treatments. However, attempts to increase the duration of triple therapy, thus prolonging the exposure to antibiotics, have not resulted in a substantial benefit. There is therefore a clear need for novel therapeutic strategies.

## 3. A Paradigm Shift to Improved Efficacy

*H. pylori* is a major human pathogen which causes a serious, transmissible, infectious disease leading to significant morbidity. However, in contrast to what is common practice in other bacterial infections (where selection of the optimal therapy is usually based on susceptibility testing), first-line therapies against *H. pylori *are only prescribed empirically. This implicates that new regimens should be properly optimized (in terms of dosage, duration of treatment, dosing intervals, and local antimicrobial resistance pattern) before their introduction in clinical practice. Moreover, resolution of the infection should be always confirmed, preferably by using a noninvasive test, providing clinicians with a reliable measure of the local drug resistance. More intuitively, optimal eradication of *H*. *pylori* has to follow two golden rules: (1) always choose the best available first-line treatment (i.e., the one that works best locally) and (2) always confirm the success of therapy by posttreatment testing and retreat patients who fail to eradicate *H. pylori*. Adoption of these two rules will guarantee for patients the best chance to be treated, with the minimum cost in terms of treatment-related adverse events and will create a useful feedback for practicing clinicians, which will prevent them from prescribing locally unacceptable regimens. 

Current approach to treatment of *H. pylori* infection is challenged by the declining efficiency of standard first-line therapies, leading to increasing need for second-line (or more) treatment courses. Paradoxically, since the initial developments in the field, infection from *H. pylori* has been approached by the digestive disease community (and not by experts in infectious diseases), as any other gastrointestinal disease (e.g., inflammatory bowel disease or irritable bowel syndrome): in the absence of an optimal treatment, the best available therapies are offered in sequence. On the contrary, for most common infections, treatment success is expected to be near 100% (i.e., ≥95%). It becomes clear that a “paradigm shift” (i.e., a change from one way of thinking to another) is necessary in order for the field to move forward [39]. Indeed, several authorities have proposed that infection with *H. pylori* should be approached and treated as any other bacterial infectious disease [40–42]. This implicates, as a general rule, that clinicians should prescribe therapeutic regimens that have a per protocol (PP) eradication rate of at least 90% (grade B level) and probably at least 95% (grade A level), in keeping with the existing practice in the field of other common bacterial infectious diseases [40, 41]. 

Development of secondary resistance (i.e., as the result of failed therapy) is largely responsible for the decline in eradication rates. Owing to this conception treatment of *H. pylori* infection is becoming a hit or miss process aiming to decrease the number of eradication failures as much as possible. As stated in the present paper, infection with *H. pylori* should be treated as any other infectious disease, and, thus, ideally, a regimen should be based on pretreatment drug susceptibility testing. In spite of this, routine use of endoscopy is not feasible and not well tolerated by all patients. Moreover, the high economic burden related to this procedure together with the disappointing results often observed in vivo by  following in vitro susceptibility is largely limiting cost efficacy of culture-guided therapy. On the contrary, enhancement of the eradication rate to values approaching 90% by adopting novel and possibly less expensive eradication strategies seems to represent a fascinating alternative.

 In recent years, promising new treatment strategies have been proposed and largely validated in some countries and are likely to represent the recommended first-line therapies in the near future [42]. Emerging first-line treatments achieving high eradication rates of 90% or more (PP analysis) are discussed below. However, it should be noted that eradication rates reported further in this paper may be prone to wide geographic variability secondary to critically important differences in the local background rates of antibiotic resistance. As empiric treatments are given without antimicrobial susceptibility testing, the choice of an empiric therapy should rely on knowledge that the combination is successful in the local population.

## 4. Emerging First-Line Treatments with a Per-Protocol Eradication Rate Exceeding 90%

### 4.1. Sequential Therapy

One recent innovation postulated as an alternative to standard triple therapy is sequential treatment, which involves a simple dual regimen including a PPI plus amoxicillin for the first 5 days followed by a triple regimen including a PPI, clarithromycin and tinidazole for the following 5 days [43]. It represents the most extensively evaluated novel therapeutic strategy including 5 comparative meta-analyses and one pooled data analysis reporting on its efficacy and safety profile [43–48]. 

In the most recent meta-analysis of 15 randomized studies (published until May 2009, including 3346 patients), sequential therapy has been demonstrated to be superior to legacy triple therapy for the eradication of *H. pylori *(91.7%, 95% Confidence Interval (CI): 90–93% versus 76, 7%, 95% CI: 75–79%, ITT analysis)  [43]. Interestingly, this regimen demonstrated ITT cure rates higher than 90% (grade B), even in countries with a high prevalence of resistance to clarithromycin, demonstrating higher performance (versus standard triple regimen) to eradicate clarithromycin-resistant strains [42]. In the meta-analysis by Gisbert et al., 41 out of 55 (75%) clarithromycin-resistant strains (4 studies) were eradicated after exposure to sequential therapy [43], although the total number with clarithromycin resistance in the included studies is still low for definite conclusions to be drawn. Similarly, the sequential regimen has been suggested as superior to legacy triple therapy in patients with metronidazole resistance [43, 48]. On the other hand, and despite this increased efficiency (in comparison with standard therapies) against sensitive and monoresistant strains, the performance of the sequential regimen seems to be dramatically compromised in the presence of dual antibiotic resistances (clarithromycin and imidazole) [49, 50]. Although the working mechanisms of the improved efficacy of the sequential regimen remain to be fully elucidated, some hypotheses may be put forward. It has been speculated that the disruption of the bacterial wall caused by amoxicillin could prevent the development of efflux channels for clarithromycin, which are known to rapidly transfer the drug out of the bacterial cell preventing the binding to the ribosome. However, according to another hypothesis, the improved effect with sequential therapy may be not attributed to the sequential administration itself; the bacteria may be simply “fulminated” by the larger number of antibiotics (3 together) to which the organism is exposed [51–53]. In accordance with this last scenario, concurrent administration of the same 3 antibiotics for a longer period of 7–10 days (i.e., the concomitant therapy, discussed further in this paper) has been shown to confer an acceptable eradication rate (89% by PP analysis and 87% by ITT analysis) when prescribed in a setting of high clarithromycin resistance (20%) where sequential regimen has been previously proved to be ineffective (cure rate 76%) [54, 55]. This data may represent preliminary, although indirect, evidence that sequential administration is probably more complicated than really necessary. 

Indeed, a major shortcoming for the use of the sequential regimen is its complexity. Although adherence to treatment was excellent in the context of clinical trials, requiring the patient to switch from a dual to a triple therapy at midpoint could inherently interfere with compliance, if this regimen is prescribed in a real clinical practice setting [56–60]. Nonetheless, almost all studies proposing sequential therapy have been conducted in Italy. Importantly, in contrast to the initial studies showing a mean overall performance approaching 90%, more recent studies conducted outside this country have shown a tendency towards lower eradication rates; in particular when dual antibiotic resistance is present [55, 61–68]. Further validation is therefore necessary before this regimen can be considered for widespread recommendation in clinical practice.

### 4.2. Nonbismuth Quadruple (Concomitant) Therapy

The concomitant regimen involves the concurrent administration of all three antibiotics used in first-line triple therapies (amoxicillin, clarithromycin, and metronidazole) given together with a PPI, all twice daily, for at least 10 days [50, 69]. This regimen is not completely novel; it has been previously evaluated with shorter durations of administration (3–7 days), in studies published between 1998 and 2002, allowing for high eradication rates (89–94% on ITT analysis) [70, 71]. It reappears nowadays as a 10-day regimen leading to eradication rates exceeding 90% on ITT analysis [50, 72]. In contrast to the sequential regimen, which has been developed and mostly evaluated in Italy, concomitant therapy has been tested in a wider range of geographical areas (including Japan, Germany, Colombia, Taiwan, and Greece) [42]. The ideal duration of administration remains an issue as direct comparisons between variable durations of treatment (e.g., 5 days versus 7 days versus 10 days) are lacking. However, one can speculate that, due to the increased antibiotic resistance rates, 3- and 5-day concomitant regimens may not be suitable today [67]. Interestingly, in a pilot study, the combination of sequential and concomitant therapies given for 14 days (hybrid therapy, PPI and amoxicillin for 7 days followed by PPI and all three antibiotics for another 7 days) achieved impressively high eradication rates (99% and 97% on PP and ITT analysis, resp.) (grade A level)  [73]. 

In Greece, a country with high resistance rates to both clarithromycin and metronidazole (>20% for clarithromycin and >40% for metronidazole), concomitant therapy has been introduced since the beginning of 2009 achieving excellent therapeutic results with cure rates of 91.6% on ITT and 94.5% on per PP analysis (grade B) [74]. It seems that concomitant therapy eradicates more than 60% of double-resistant **H. pylori ** strains and the vast majority of sensitive and monoresistant strains, thus preventing the emergence of secondary resistance [75]. At the same time, means of tolerability and safety profile are reported to be excellent and comparable to those obtained with standard triple therapy [74, 75]. 

A main advantage of the concomitant (versus sequential) therapy may be represented by its suitability for patients with dual resistance to antibiotics. Indeed, in a comparative study by Wu et al., patients with resistance to both clarithromycin and metronidazole had significantly lower eradication rates after sequential therapy (present versus absent: 33.3% versus 95.1%; *P*-value < 0.0001), but not after concomitant therapy (present versus absent: 75.0% versus 92.4%; *P*-value = 0.22) [50]. However, it should be noted that this study was conducted in Taiwan where the rate of antibiotic resistance is very low and even standard triple therapy is currently yielding excellent eradication rates [76]. A comparison study conducted across a broad range of patients and with a high prevalence of antibiotic-resistant *H. pylori* strains would be therefore much appreciated in order to definitely solve the issue of concurrent versus sequential administration; these two emerging treatment options seem to represent the main competitors likely to replace triple therapy in the foreseeable future.

### 4.3. Bismuth-Containing Quadruple Therapy

This regimen is mainly used as second-line treatment when legacy triple therapy fails, but also as an alternative first-line treatment option in regions with a high incidence of resistance to clarithromycin [77]. Other than working independently from resistance to clarithromycin, the main advantage of this regimen is represented by the limited clinical impact of metronidazole resistance which can be largely overcome by increasing the dose of metronidazole and duration of treatment. Considering that resistance to metronidazole in most countries is currently exceeding 10%, the daily dose of metronidazole prescribed should be approximately 1500 mg (3 × 500 or 4 × 400 mg in England) in order for maximal cure rates to be obtained. 

Historically, in an early meta-analysis, first-line use of a bismuth-containing quadruple therapy (BQT) yielded high eradication rates (grade A or B level) [78]. These encouraging results have been mainly attributed to the efficacy against metronidazole-resistant strains, which overcome the eradication achieved with standard triple therapy over clarithromycin-resistant strains [23, 79]. However, according to a more recent meta-analysis, performance of both BQT and standard regimen was suboptimal (78.3% versus 77% on ITT analysis) [23]. In our country, BQT has been mainly used as a second-line therapy leading to rather contradictory results [28, 80]. In the only study where BQT has been used as first-line treatment and compared to standard triple therapy, both given for 10 days, results were disappointing (eradication rates 65% versus 78% on ITT analysis), whereas a higher incidence of adverse events was observed among patients receiving BQT [29]. 

A practical issue limiting the use of BQT is the absence of HCL tetracycline in some countries and the unavailability of bismuth salts in some other. Substitution of tetracycline with doxycycline or amoxicillin, in order to overcome this problem, was associated with rather disappointing results [81, 82]. On the contrary, high success rates were reported when BQT was used in the form of one capsule containing bismuth with both the antibiotics (metronidazole plus tetracycline). Three of these monocapsules are given four times daily in combination with a PPI twice daily for 10 days; this bismuth-based triple therapy monocapsule represents a patient-friendly formulation which is aimed to increase compliance to treatment [83, 84]. Currently, two of these monocapsules are available in the market, Helidac (USA) containing a lower dose of metronidazole (1 gr instead of 1.5 gr) and Pylera (USA and Europe) containing a lower dose of Tetracycline (1.5 gr instead of 2 gr), as compared to the classic BQT. These therapies seem to overcome *H. pylori* resistance to metronidazole since they achieve high eradication rates, reportedly exceeding 90% [85–88].

### 4.4. Alternative First-Line Therapies

In recent years, some authorities have proposed the use of levofloxacin, instead of clarithromycin, as the main compound of first-line treatments, achieving contradictory results [55, 76, 89]. Indeed, eradication rates with the use of levofloxacin-based triple therapy have been varying from 72% to 90% (ITT analysis), and this regimen has been suggested as an efficient alternative in settings of clarithromycin resistance exceeding 15%–20% and quinolone resistance less than 10% [90]. Interestingly, a novel levofloxacin-based sequential regimen was more effective than the standard clarithromycin-based sequential regimen in a setting with a high clarithromycin resistance rate (20%) where the latter has yielded suboptimal eradication rates (<80% in ITT) [89]. However, it should be noted that primary levofloxacin resistance in the study was very low (3.7%), and therefore these results may be difficult to reproduce in geographical areas with higher rates of quinolone resistance. Rapid development of resistance, as well as the high incidence of adverse events, represents further drawbacks concerning the use of levofloxacin in first-line treatment [91–96]. For these reasons, levofloxacin-based regimens are generally considered more suitable for use as second-line treatments or as salvage therapies [90, 97–101].

## 5. Therapeutic Algorithm of *H. pylori * Infection in Clinical Practice

The recommended regimens for *H. pylori *therapies are summarized in Table 1. Choice of the optimal, among these regimens, has to follow the rule of what works best locally; this should be based on the knowledge of the local *H. pylori *resistance pattern and the continuous evaluation of treatment outcomes (posttreatment testing) in clinical practice [42, 102]. For 5–10% of patients, even the emerging first-line therapies, described in this paper, are expected to be unsuccessful. In these cases, empiric use of a levofloxacin-based triple therapy seems to represent a reasonable option if local resistance to this antibiotic does not exceed 10% [102–104]. Alternatively, a bismuth-based quadruple therapy can be used for 14 days, since this regimen seems to overcome, at least partially, resistance to metronidazole [105–107]. The old dual regimen of a PPI plus amoxicillin given twice daily (and abandoned because of low eradication rates (<50%)), returns nowadays with the administration of higher doses of both drugs (PPI × 3 and amoxicillin 1000 mg × 3). With the new dosing scheme this dual regimen can be used as salvage therapy in areas with high resistance rates to levofloxacin [108]. The small minority of patients (<1%) with refractory *H. pylori *infection to both first- and second-line treatments have to be referred for antibiotic susceptibility testing in order for third-line therapies to be instituted [104, 109]. Alternatively, rifabutin-based or furazolidone-based therapies can be employed for the treatment of refractory *H. pylori *infection [110, 111].

Importantly, most of the aforementioned emerging first-line therapies have not been incorporated into international guidelines so far [25, 77], although this does not seem to be too far away according to more recent recommendations [112]. However, there is still work to be done in order for these novel regimens to be sufficiently validated and therefore possibly recommended as first choice therapies ushering in a new era of anti-*H. pylori *treatment. 

## Figures and Tables

**Table 1:** Recommended regimens for *Helicobacter pylori* therapy.

Treatment	Regimen
First-line treatments	
Sequential therapy	A 5 d dual therapy with a PPI (standard dose, b.i.d.) and amoxicillin (1 g, b.i.d.) followed by a 5 d triple therapy with a PPI (standard dose, b.i.d.), clarithromycin (500 mg, b.i.d.), and metronidazole (500 mg, b.i.d.)
Concomitant therapy	A PPI (standard dose, b.i.d.), clarithromycin (500 mg, b.i.d.), amoxicillin (1 g, b.i.d.), and metronidazole (500 mg, b.i.d.) for 7–10 d
Hybrid therapy	A 7 d dual therapy with a PPI (standard dose, b.i.d.) and amoxicillin (1 g, b.i.d.) followed by a 7 d quadruple therapy with a PPI (standard dose, b.i.d.), amoxicillin (1 g, b.i.d.), clarithromycin (500 mg, b.i.d.), and metronidazole (500 mg, b.i.d.)
Bismuth-containing quadruple therapy	A PPI (standard dose, b.i.d.), bismuth (standard dose, q.i.d.), tetracycline (500 mg, q.i.d.), and metronidazole (500 mg, t.i.d.) for 10–14 d

Second-line/Salvage treatments	
Levofloxacin-based triple therapy	A PPI (standard dose, b.i.d.), levofloxacin (500 mg, b.i.d.), and amoxicillin (1 g, b.i.d.) for 10 d
Bismuth-containing quadruple therapy	A PPI (standard dose, b.i.d.), bismuth (standard dose, q.i.d.), tetracycline (500 mg, q.i.d.), and metronidazole (500 mg, t.i.d.) for 14 d
Standard triple therapy^∗^	A PPI (standard dose, b.i.d.), amoxicillin (1 g, b.i.d.), and clarithromycin (500 mg, b.i.d.) for 14 days
Levofloxacin-based sequential therapy^∗∗^	A 5 d dual therapy with a PPI (standard dose, b.i.d.) and amoxicillin (1 g, b.i.d.) followed by a 5 d triple therapy with a PPI (standard dose, b.i.d.), levofloxacin (250 mg, b.i.d.), and amoxicillin (1 g, b.i.d.)
Amoxicillin-based dual therapy (high dose)^∧^	A PPI (high dose, t.i.d) and Amoxicillin (1 g, t.i.d.) for 14 days
Rifabutin-based triple therapy^∧^	A PPI (standard dose, b.i.d.), rifabutin (150 mg b.i.d.), and amoxicillin (1 g b.i.d.) for 14 d
Furazolidone-based quadruple therapy^∧^	A PPI (standard dose, b.i.d.), tripotassium dicitratobismuthate (240 mg, b.i.d.), furazolidone (200 mg, b.i.d.), and tetracycline (1 g, b.i.d.)

^∗^Employed after antibiotic susceptibility testing; ^∗∗^regimen under evaluation; ^∧^regimen usually employed as third-line therapy; PPI: proton pump inhibitor.
